# Assessment of microexon-associated junction usage in Parkinson’s disease and spinocerebellar Ataxia type 3

**DOI:** 10.3389/fnmol.2026.1872346

**Published:** 2026-09-17

**Authors:** Kira Mehnert, Jeannette Hübener-Schmid, Jakob Admard, Vladislav Lysenkov, German Demidov, Chrisovalantou Huridou, Jonasz Jeremiasz Weber, Olaf Riess, Nicolas Casadei

**Affiliations:** 1 Institute of Medical Genetics and Applied Genomics, University of Tübingen, Tübingen, Germany; 2 Department of Life Sciences, Reutlingen University, Reutlingen, Germany; 3 DFG NGS Competence Center Tübingen (NCCT), University of Tübingen, Tübingen, Germany; 4 Department of Human Genetics, Ruhr University Bochum, Bochum, Germany

**Keywords:** exon skipping, microexons, neurodegeneration, Parkinson’s disease, spinocerebellar ataxia type 3

## Abstract

**Background:**

Microexons are short exonic sequences of 3–30 nucleotides that undergo alternative splicing and have been implicated in several neurodevelopmental disorders. However, their contribution to neurodegenerative diseases remains insufficiently characterized.

**Methods:**

We analyzed microexon-associated junction usage in the frontal cortex of Parkinson’s disease (PD) patients and in the cerebellum of Spinocerebellar Ataxia Type 3 (SCA3) patients using deep RNA-Sequencing of human postmortem brain tissue and corresponding SH-SY5Y cellular models. Microexon-associated junction usage was quantified based on splice junction reads derived from STAR alignments, followed by statistical testing with false discovery rate (FDR) correction for multiple testing and visualization using Sashimi plots. Selected candidate events in the cellular datasets were assessed by PCR.

**Results:**

Across all datasets, 19 microexon-associated junction candidate events were identified but did not survive FDR correction. Human brain tissue exhibited substantially more detectable microexons compared to cellular models, which may reflect differences in splicing complexity between differentiated tissue and neuroblastoma-derived cells. While visual inspection suggested that effect sizes were generally small and variable across samples, the majority of microexon-containing genes were expressed in both systems. PCR confirmed detection of the selected microexons but did not validate differences in microexon-associated junction usage between experimental groups.

**Conclusion:**

Our findings provide an overview of microexon landscapes in PD and SCA3 and an exploratory statistical analysis. Although large and consistent disease-specific patterns of microexon-associated junction usage were not observed, our findings suggest that microexon regulation in neurodegeneration may involve subtle and context-dependent alterations that warrant further investigation in larger cohorts.

## 1 Introduction

Alternative splicing is an important post-transcriptional regulatory mechanism in the human genome, particularly in the context of disease. It generates multiple mRNA isoforms from a single gene (Cui et al., 2017).

As demonstrated in previous studies, exons consisting of only a few nucleotides, known as microexons, encounter challenges during the alternative splicing process, leading to an increased frequency of exon skipping (Ustianenko et al., 2017). The initial identification of microexons was made in 1985 in the *UBX* gene of the fruit fly, *Drosophila melanogaster* (Beachy et al., 1985). Since then, a considerable number of additional microexons—defined as short exonic sequences comprising three to 30 nucleotides—have been the subject of characterization. The prevalence of microexons in neuronal tissue has been well documented. Genes containing microexons have been found to be particularly enriched in pathways involved in synaptic function and neuronal development, highlighting their central role in the nervous system (Irimia et al., 2014). The inclusion or exclusion of microexons in mRNA transcripts has been demonstrated to exert a substantial influence on protein function, consequently impacting neuronal connectivity and signaling pathways. This precise regulation is essential for maintaining the delicate balance necessary for normal brain function (Li et al., 2015). Recent studies have linked aberrant microexon splicing to various neurological disorders, including autism spectrum disorders (ASDs), schizophrenia, and epilepsy. Research has demonstrated that misregulated neuronal microexons contribute to the pathogenesis of several neurodevelopmental disorders, particularly ASD, by affecting genes involved in synaptic transmission and neuronal development (Irimia et al., 2014).

Whether similar alterations occur in neurodegenerative diseases remains largely unexplored.

Increasing evidence suggests that disturbances in RNA metabolism and alternative splicing contribute to the pathogenesis of several neurodegenerative disorders (Nik and Bowman, 2019). In Parkinson’s disease (PD), altered alternative splicing of the *SNCA* gene and disease-associated splicing signatures have been reported, suggesting a contribution of splicing dysregulation to disease pathogenesis (Nikom and Zheng, 2023). In spinocerebellar ataxia type 3 (SCA3), alternative splicing of the *ATXN3* gene has been implicated in disease pathogenesis, and *ATXN3* transcript diversity appears to be particularly relevant in cerebellar tissue (Raposo et al., 2023). Given the specific splicing requirements of microexons, the alterations in alternative splicing reported in PD and SCA3 raise the possibility that microexon-associated junction usage may also be altered in these neurodegenerative disorders. The objective of this study was to investigate microexon-associated junction usage in PD and SCA3.

## 2 Materials and methods

### 2.1 RNA-sequencing

The human RNA-Sequencing (RNA-Seq) datasets were obtained from previously published studies (Haas et al., 2022; Hentrich et al., 2020). The PD dataset consisted of postmortem frontal cortex samples from six PD patients and six controls. The patient group included three female and three male individuals, with patient ages ranging from 67 to 88 years and control ages from 60 to 87 years. The SCA3 dataset comprised cerebellar samples from six SCA3 patients and six controls. The SCA3 cohort was exclusively male, with patient ages ranging from 56 to 77 years and control ages from 31 to 79 years.

Cellular models were generated using the human neuroblastoma cell line SH-SY5Y. For the PD model, SH-SY5Y cells were transfected with synuclein constructs, including the aggregation-prone SynT construct and GFP construct, as previously described (Lázaro et al., 2014). For the SCA3 model, cells were transfected with pEGFP-ATXN3 plasmid constructs containing either 15 or 148 CAG repeats, as previously described (Gonsior et al., 2021), or with pcDNA3.1 ataxin-3-myc constructs containing 15 or 148 CAG repeats (BioCat, Heidelberg, Germany) using the Lipofectamine 3,000 Transfection Reagent according to the manufacturer’s protocol for 72 h. To ensure reproducibility, four independent biological replicates of the cell models were generated.

The concentration of RNA was measured using the Qubit Fluorometric Quantitation and RNA Broad-Range Assay (Thermo Fisher Scientific). RNA integrity was assessed using the Fragment Analyzer 5,300 and the Fragment Analyzer RNA Kit (Agilent Technologies) with all samples showing RNA Integrity Numbers (RINs) > 7. For library preparation, the mRNA fraction was enriched using poly(A) capture from 100 ng of total RNA using the New England Biolabs (NEB)Next Poly(A) mRNA Magnetic Isolation Module (NEB). Subsequently, libraries were prepared using the NEBNext Ultra II Directional RNA Library Prep Kit for Illumina and NEBNext UDI unique molecular identifiers (NEB) following the manufacturer’s instructions. To minimize technical batch effects, library preparations were performed using the liquid handler Biomek i7 (Beckman). The library molarity was determined by measuring the library size (approximately 400 bp) using the Fragment Analyzer 5,300 and the Fragment Analyzer DNA HS NGS Fragment Kit (Agilent Technologies) and the library concentration (>2 ng/μl) using Qubit Fluorometric Quantitation and the dsDNA High Sensitivity Assay (Thermo Fisher Scientific). The libraries were denatured according to the manufacturer’s instructions, diluted to 150 pM and sequenced as paired-end 100 bp reads on an Illumina NovaSeq 6000 (Illumina). Sequencing was performed to achieve a depth of approximately 20 million clusters per sample.

Read quality of RNA-Seq data in FASTQ files was assessed using ngs-bits (master branch, commit g2b2373e8, January 2025) to identify sequencing cycles with low average quality, adaptor contamination, or repetitive sequences from PCR amplification. Reads were aligned using STAR (v 2.7.11b; Dobin et al., 2013) to the GRCh38 reference genome and alignment quality was analyzed using ngs-bits and visually inspected in the Integrative Genomics Viewer (v2.15.4). Normalized read counts for all genes were obtained using Subread (v2.0.4).

### 2.2 Microexon analysis

To analyze microexon-associated junction usage, RNA-Seq data were aligned to the reference genome using STAR (v2.7.11b). Exons with a length between 3 and 30 nucleotides were retained. To reduce noise, only exons with at least five reads in 50% of the samples and at least five splice-junction reads in a minimum of four samples were included.

A custom Python script (v3.12.5, Python Software Foundation, Wilmington, USA) was used to merge raw exon counts with splice-junction read data and to annotate microexon-associated junction events (DOI:10.5281/zenodo.22179987).

Junction usage was then defined as the ratio of junction-spanning reads to total gene expression for the corresponding gene


$$U_{g,s}=\frac{J_{g,s}}{C_{g}}$$

where *J_g,s_* represents the number of reads spanning junction *s* in gene *g*, and *C_g_* denotes the raw gene expression count of gene g. This metric provides a relative measure of splice junction usage normalized to overall gene expression within each sample. Raw gene counts were used to maintain proportional scaling between junction-level and gene-level read counts derived from the same alignment output. Additional library size normalization was not required, as counts were interpreted relative to the total gene count for each sample, as described in a previous study (Segers et al., 2026). The approach does not represent a classical percent-spliced-in (PSI) metric but enables consistent comparison of relative junction usage across heterogeneous datasets. Following an initial PSI-based analysis using VAST-DB, microexon-associated junction usage was used as the primary measure for the subsequent analysis of this study.

### 2.3 Statistics

Statistical analyses were performed using RStudio (v2024.09.0 + 375, Posit Software PBC, Boston, USA). *p*-values were calculated using the Mann–Whitney-U-test. The distribution of *p*-values was assessed across datasets to evaluate the overall behavior of the statistical testing. False discovery rate (FDR)-adjusted *p*-values were calculated for all tested junction events using the Benjamini–Hochberg method. Volcano plots were generated using nominal *p*-values (*p* < 0.05) and the mean difference in microexon-associated junction usage (|Δ-mean| ≥ 0.01) to identify candidate microexon-associated junction events. As no events remained statistically significant after FDR correction, nominal *p*-values were used solely to prioritize candidate events for exploratory follow-up analyses. Candidate events were visualized using Sashimi plots generated with the Integrative Genomics Viewer (IGV, version 2.17.4, Broad Institute, Cambridge, USA) using the *Homo sapiens* reference genome (GRCh38/hg38). To enhance plot clarity, a minimum junction coverage of five was applied. Candidate events selected from the cellular RNA-Seq analysis were further assessed by PCR. Genes harboring nominally significant microexon-associated junction events were analyzed using Ingenuity Pathway Analysis (IPA; v01.23.01, QIAGEN, Hilden, Germany) to explore associated canonical pathways and biological functions.

### 2.4 PCR validation

500 ng of RNA was reverse-transcribed into cDNA using the QuantiTect Reverse Transcription Kit (QIAGEN) and according to the manufacturer’s instructions. For each target gene, a primer panel was designed (Supplementary Table S1) using Primer-BLAST (Ye et al., 2012): a flanking primer pair located in the exons upstream and downstream of the microexon, amplifying both inclusion and skipping isoforms in a single RT-PCR reaction; a qPCR primer pair specific for the microexon-inclusive isoform; a qPCR primer pair specific for the microexon-skipping isoform; and a reference qPCR primer pair located in constitutive exons used for normalization. Quantitative PCR was performed using the QuantiTect SYBR Green PCR Kit (QIAGEN) on a Roche LightCycler 480, according to the manufacturer’s instructions. A single melting peak was confirmed for each assay as a quality control, alongside no-template and no-RT controls. Relative expression was calculated using the 2^-ΔΔCp method, normalized to the gene-specific reference amplicon, and expressed relative to the control group mean. Technical replicates were averaged per sample prior to analysis. Group differences were assessed using Welch’s t-test on ΔCq values, with Benjamini-Hochberg correction across comparisons.

### 2.5 Expression analysis

To assess the expression of genes harboring filtered human microexons in the corresponding cellular datasets, raw gene counts obtained from subread (v2.0.4) were converted to counts per million (CPM) in R by normalizing to the total library size of each sample. Genes were categorized as expressed if their CPM was >1 in at least one cellular sample. Genes with CPM ≤ 1 in all cellular samples were classified as lowly expressed, whereas genes associated with filtered human microexons but showing no expression in any cellular sample were considered not expressed. Duplicate gene entries were removed prior to analysis to retain unique gene identifiers. As functional enrichment analyses are performed at the gene level, genes harboring filtered human microexons were used for downstream analyses. To further explore functional similarities between genes shared across human and cellular datasets, as well as genes unique to human samples, gene lists were analyzed using g: Profiler (Kolberg et al., 2023) to perform functional enrichment analysis of Gene Ontology Molecular Function (GO: MF) terms and Reactome pathways.

## 3 Results

Although none of the identified candidate microexon-associated junction events remained statistically significant after false discovery rate correction, top candidates were further explored in downstream analyses because of the exploratory nature of this study. In the frontal cortex of human postmortem PD patients, 444 microexons passed the filtering criteria. Seven microexon-associated junction events displayed nominally increased junction usage (*ANKRD6*, *CHPT1, TMEM120A*, *TMEM144,* and *TOP3A*, as well as two independent junctions corresponding to the same microexon in *BLOC1S6*), while two microexon-associated junction events showed nominally decreased junction usage (*EGFL7* and *TENM4*; Figure 1A). In the PD cell model, 361 microexons were analyzed, of which five candidate microexon-associated junction events were identified. Two candidate events (*HSD11B1L* and *WDR62*) suggest an increased junction usage, whereas three candidate events (*HP1BP3*, *MRPL33*, and *PABPC1L*) suggest decreased junction usage (Figure 1C). In the cerebellum of patients with SCA3, 1,520 microexons were analyzed, suggesting three microexon-associated junction candidate events: two suggested a potential increased junction usage (*ACTN1* and *NDUFAF5*) and one suggested a potential decreased junction usage (*AQP7*; Figure 1B). In the SCA3 cell model, 433 microexons were evaluated, identifying two candidate microexon-associated junction events with nominally increased junction usage (*CACNB4* and *DPH5-DT*; Figure 1D). Detailed results for all datasets, including all filtered microexons, are provided in the Supplementary Tables S2–S5 and the Supplementary Figure S1.

**Figure 1:**
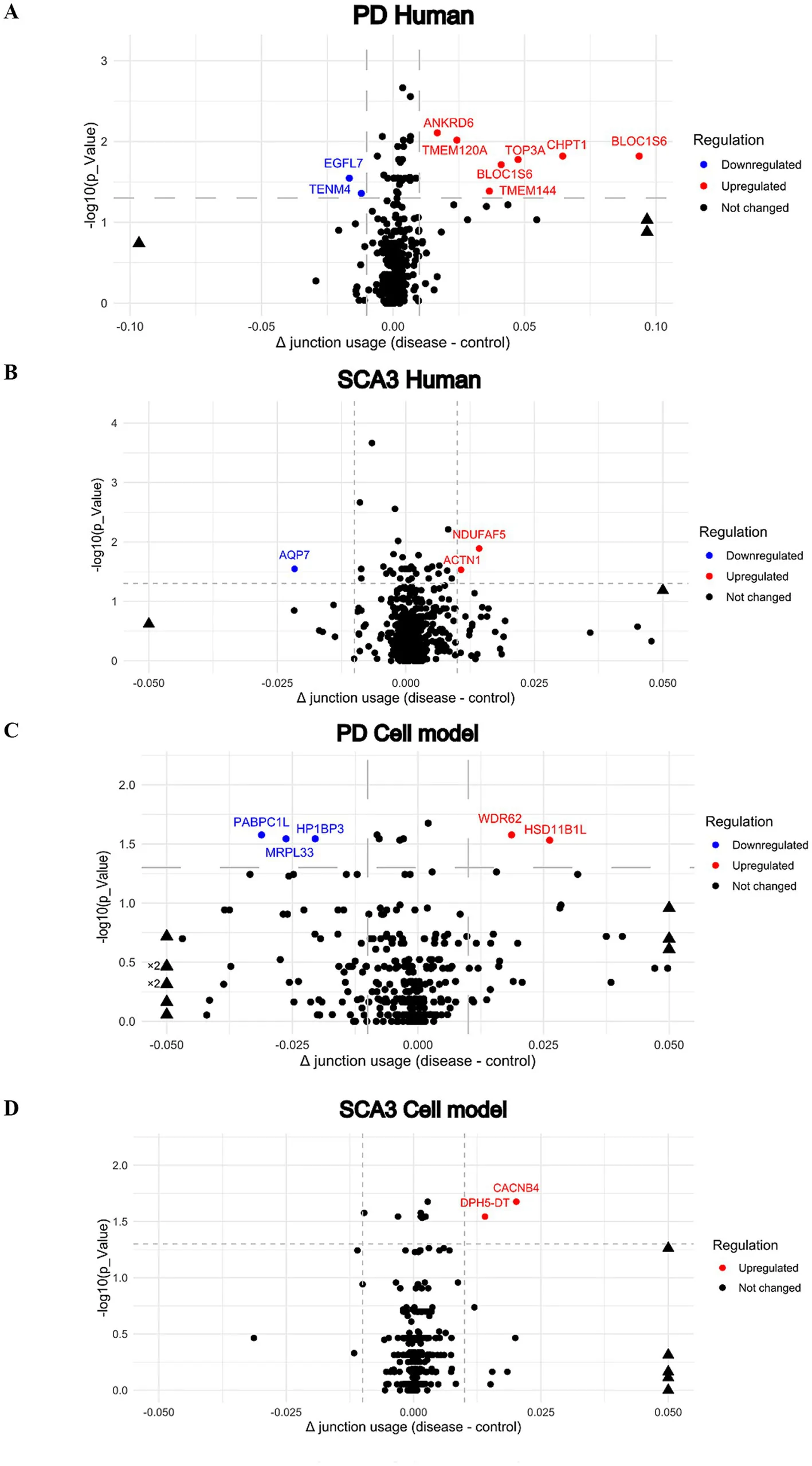
Volcano plots of the Parkinson disease (PD) and Spinocerebellar Ataxia Type 3 (SCA3) models. Volcano plots of the filtered **(A)** PD human (*n* = 6 vs. 6), **(B)** SCA3 human (*n* = 6 vs. 6), **(C)** PD cells (*n* = 4 vs. 4) and **(D)** SCA3 cells (*n* = 4 vs. 4) datasets. *P*-values were calculated using Mann–Whitney-U-test with a threshold of *p* < 0.05. Δ-junction usage was calculated as described in the Materials and Methods section with a threshold of ≥ 0.01. Colored data points indicate nominally significant events. None of the events remained statistically significant after false discovery rate (FDR) correction for multiple testing. Triangles indicate events outside the displayed axis range. These events were not excluded from the statistical analysis. Two closely spaced microexon start positions were detected for *ACTN1*. The alternative microexon start site observed in *EGFL7* is consistent with previously reported exon boundaries (Nikolic et al., 2010).

Although statistical analysis identified potential candidate microexon-associated junction events, visualization using IGV Sashimi plots did not consistently reveal clear differences in microexon-associated junction usage between affected and control groups. Read coverage varied substantially across samples, and visually apparent differences were not uniformly aligned with disease status. Modified Sashimi plots for all candidate events are provided in the Supplementary Figures S2–S19. The biological relevance of a subset of detected microexon events was further investigated by RT-PCR and qPCR in the cellular RNA-seq datasets. These candidates were chosen due to their high gene expression, significant levels of microexon skipping or inclusion, and clear microexon annotation, which increased the chances of successful primer design and amplification. The focus was on six genes (PD model: MEF2C, MRPL33, SORBS1; SCA3 model: APBB1, PHB2, MEF2A). For four of these genes (MEF2C, MRPL33, MEF2A, PHB2), both microexon-containing and -skipping isoforms were successfully amplified; however, SORBS1 and APBB1 did not produce specific products and were thus excluded from further analysis. In the four assays that were technically interpretable, qPCR did not confirm any significant differences in microexon-associated junction usage between the experimental groups (Supplementary Figure S20).

To explore pathway associations of genes harboring the identified candidate events, functional pathway analysis was performed using IPA. No genes were shared between the identified pathways. The pathways comprised a broad range of biological functions, including mitochondrial networks and autophagy-related processes.

To further assess the comparability between human and cellular models, we evaluated the expression of all genes harboring filtered human microexons in the corresponding cellular RNA-Seq datasets. In the PD dataset, the 444 filtered microexons corresponded to 308 unique genes. Of these, 285 genes (92.53%) were expressed at >1 CPM in the cellular model, two genes (0.65%) showed low expression (≤1 CPM), and 21 genes (6.82%) were not detected (Figure 2). In the SCA3 dataset, 1,520 filtered microexons corresponded to 557 unique genes. Among these, 532 genes (95.51%) were expressed at >1 CPM in the cellular model, no genes had low expression (≤1 CPM), and 25 genes (4.49%) were not detected (Figure 2).

**Figure 2:**
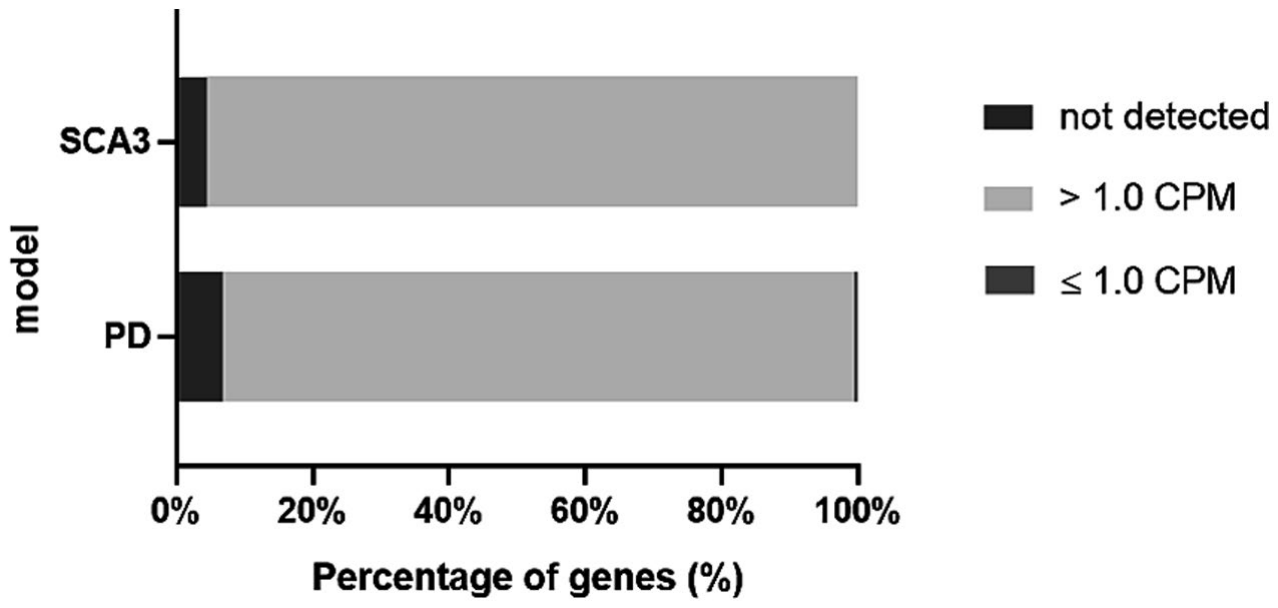
Expression status of human microexon-containing genes in the cellular models. Stacked bar plots of the human microexon-containing genes detected in the corresponding Parkinson Disease (PD) and Spinocerebellar Ataxia Type 3 (SCA3) cell datasets. Gene expression levels were classified as > 1 counts per million (CPM), ≤ 1 CPM, or not detected. Percentages were calculated relative to the total number of human microexon-containing genes identified in each dataset.

## 4 Discussion

Increasing evidence points to microexon splicing as an important regulatory component in maintaining neurological function (Irimia et al., 2014; Ustianenko et al., 2017). In the present study, we observed several nominally significant microexon-associated junction events across PD and SCA3 datasets. However, none of these events remained significant after FDR correction, indicating that the observed differences should be interpreted as preliminary and hypothesis-generating rather than confirmatory.

Our comparative analysis demonstrated a higher number of detected microexons in human brain tissue (PD: 444; SCA3: 1,520) compared to SH-SY5Y cell models (PD: 361; SCA3: 433). This discrepancy may reflect the advanced differentiation state and structural complexity of human brain tissue, which is expected to exhibit a more specialized splicing landscape than neuroblastoma-derived cell lines. SH-SY5Y cells, while widely used in neurobiological research, retain characteristics of their tumor origin and represent a partially differentiated neuronal model, which may limit their ability to fully recapitulate mature neuronal splicing programs (Kovalevich et al., 2021). Consistent with this, expression analysis revealed that a small subset of genes harboring human microexons was not detectable in the corresponding cellular datasets (~ 4–7%), while the majority (>92%) were expressed above 1 CPM. Together with the relatively small sample size of the cellular model, these observations indicate that the SH-SY5Y findings should be regarded as exploratory and interpreted with appropriate caution.

Within the set of genes shared between human and cellular datasets, several could be assigned to functionally related pathways. In the PD datasets, genes such as *ANK2* and *AP2M1* were associated with membrane trafficking and vesicle transport. In contrast, genes uniquely detected in the human PD dataset could not be clearly assigned to shared pathways, indicating a more heterogeneous functional distribution. Similarly, in the SCA3 datasets, shared genes including *PRKAG2* and *DYNC1H1* were linked to pathways related to cellular maintenance and intracellular transport. In contrast, genes detected exclusively in the human SCA3 dataset, such as *CACNA1A* and *CACNA1E*, were associated with presynaptic processes and calcium channel activity. Given the known role of calcium homeostasis in neuronal function, this may indicate biological processes that are not fully captured in the cellular model.

Although several events showed nominally significant differential junction usage prior to multiple-testing correction, IGV visualization did not consistently reveal clear differences in microexon-associated junction usage between disease and control groups, indicating that the observed differences were subtle and variable across samples. The PCR analysis provided technical support for the detection of the identified microexon-associated junction events. However, the lack of validated group-specific differences limits conclusions regarding their biological relevance. Furthermore, the events identified in the human datasets were not independently validated by PCR. Therefore, these findings should be interpreted with caution and require further validation.

To further assess the biological relevance of these findings, we examined whether the affected genes have previously been described in the context of neurodegeneration. Several of these genes are involved in biological processes relevant to neuronal function and neurodegeneration. In the PD datasets, *BLOC1S6* has been reported to be upregulated in PD patients (Kim and Daadi, 2019), and variants in *TENM4* have been discussed as potential risk factors (Pu et al., 2020), consistent with a potential association with disease pathology. In addition, *HSD11B1L* has been proposed as a candidate gene for PD (Zhao et al., 2025), while *PABPC1L* has been implicated in neurodegenerative phenotypes (Kong et al., 2022). Genes such as *TOP3A* and *TMEM144* have been linked to neurological conditions (Wang et al., 2025a; Wang et al., 2025b), while *TMEM120A* has been implicated in neuronal processes, including nociception (Kang and Lee, 2024). Additional genes identified in these datasets, including *ANKRD6*, *EGFL7*, *CHPT1*, *HP1BP3*, *WDR62* and *MRPL33*, are involved in cellular functions relevant to neuronal integrity and signaling (Liu et al., 2018; McDonald and Schmidt, 2024; Neuner et al., 2019; Theiss et al., 2022; Tissir et al., 2002; Xu et al., 2018). Importantly, although many of these genes have previously been implicated in neuronal function or neurodegenerative disease, the functional relevance of the specific microexon-associated junction events identified in the present study remains unknown. Taken together, these observations suggest that the identified genes are associated with processes related to neuronal function and, in some cases, disease-associated mechanisms, whereas the contribution of the detected microexon-associated junction events to disease pathogenesis remains to be established.

In the SCA3 dataset, the identified genes likewise were associated with processes relevant to neuronal function and disease mechanisms. *NDUFAF5* has been linked to neurological disorders associated with mitochondrial dysfunction (Massuyama et al., 2025), while *AQP7*, *ACTN1, DPH5* and *CACNB4* have been associated with cellular stress responses, cytoskeletal organization, diphthamide biosynthesis, and neuronal calcium signaling, respectively (Alsouri et al., 2024; Parker et al., 2024; Politano et al., 2025; Qi et al., 2025). These genes are associated with biological processes involved in neuronal function and homeostasis. Consequently, the identified microexon-associated junction events represent biologically plausible candidates for further investigation, although their functional significance remains unknown. Direct evidence linking these specific microexon-associated events to SCA3 pathogenesis is currently lacking. Microexons within these genes have not been characterized in detail to date, indicating that their potential functional relevance in these contexts remains largely unexplored. An initial PSI-based analysis using VAST-TOOLS did not allow reliable identification of the selected microexon-associated events. Therefore, microexon-associated junction usage was used as the primary measure in the present study.

Because the present study specifically focused on microexon-associated events rather than global splicing alterations, genome-wide splicing detection tools such as FRASER (Scheller et al., 2023) were not applied. Due to their short length and often low junction coverage, microexons are generally challenging to detect with genome-wide approaches, which may limit sensitivity. In addition, the relatively small cohort size is expected to reduce the statistical robustness of genome-wide approaches, particularly in the context of multiple testing, and is likely to limit the interpretability of such results. A targeted analysis approach was therefore applied to enable the detection of microexon-associated events under these conditions.

Several additional limitations should be considered when interpreting the findings obtained from postmortem human brain tissue. Variability in age, sex, RNA integrity, postmortem interval, batch effects, disease-associated neuronal loss, gliosis, and differences in cell-type composition may influence the observed microexon-associated junction usage. Because these factors were not explicitly controlled for in the present study, some of the observed differences may reflect changes in tissue composition rather than disease-specific alterations in microexon regulation. Future studies incorporating adjustment for relevant clinical and technical covariates as well as cell-type-aware analyses or deconvolution approaches will be important to further validate these findings.

Overall, our findings provide a descriptive characterization of microexon landscapes in human postmortem brain tissue and corresponding SH-SY5Y cellular models. Although selected events were further evaluated by PCR, the biological significance of the detected microexon-associated junction events remains to be determined. Given the absence of FDR-significant events, the exploratory nature of the SH-SY5Y model, and the limitations associated with postmortem tissue, the present findings should be regarded as hypothesis-generating. Future studies incorporating larger cohorts, deeper sequencing coverage, cell-type-aware analyses, functional characterization of candidate microexons, and more physiologically relevant neuronal models will be required to determine whether microexon dysregulation contributes to neurodegenerative disease mechanisms.

## Data Availability

The datasets presented in this study can be found in online repositories. The names of the repository/repositories and accession number(s) can be found in the article/Supplementary material.
