# Supplementary material for: Assessment of microexon-associated junction usage in Parkinson’s disease and spinocerebellar Ataxia type 3
Source: Front Mol Neurosci. 2026 Sep 17;19:1872346. doi: 10.3389/fnmol.2026.1872346 (PMC13627293; doi:10.3389/fnmol.2026.1872346)
Supplement: Supplementary file 1 [file Table_1.DOCX]

Supplementary Material

# 1 Supplementary Tables

**Supplementary Table S1.** List of primers used for the microexon validation

**Supplementary Table S2.** Filtered microexon dataset for all detected events in human PD samples

**Supplementary Table S3.** Filtered microexon dataset for all detected events in human SCA3 samples

**Supplementary Table S4.** Filtered microexon dataset for all detected events from the PD cell model

**Supplementary Table S5.** Filtered microexon dataset for all detected events from the SCA3 cell model

# 2 Supplementary Figures

**Supplementary Figure S1. junction usage of highlighted candidate microexon events.**

Violin plots illustrating the junction usage per sample (junction-spanning reads adjusted for gene expression, resulting in a dimensionless ratio) for the candidate events emphasized in the volcano plots of Figure 1, categorized by experimental contrast (PD, human frontal cortex; SCA3, human cerebellum; PD, SH-SY5Y cell model; SCA3, SH-SY5Y cell model). Each panel represents a single gene, with the relevant microexon event noted if a gene had multiple candidate events (e.g., BLOC1S6, events 1 and 2). Black dots denote individual biological replicates, while the horizontal black line indicates the median of the group. Control samples are depicted in green, and disease samples in orange. After FDR correction, none of the previously highlighted events remained significant.

**Supplementary Figure S2. modified Sashimi plots of 22 nucleotide microexon in *ACTN1* gene**

Sashimi plots generated from IGV data of the human SCA3 dataset (n = 6 vs. 6) are shown for (A) affected individuals and (B) controls. A minimum splice junction coverage of five was applied to all samples. For clarity, splice junctions were simplified by merging parallel junctions that skip or include the same microexon into a representative connecting line, with the summed number of junction reads indicated in the arc. The graphical simplification of splice junctions was performed using Inkscape.

**Supplementary Figure S3. modified Sashimi plots of 9 nucleotide microexon in *ANKRD6* gene**

Sashimi plots generated from IGV data of the human PD dataset (n = 6 vs. 6) are shown for (A) affected individuals and (B) controls. A minimum splice junction coverage of five was applied to all samples. For clarity, splice junctions were simplified by merging parallel junctions that skip or include the same microexon into a representative connecting line, with the summed number of junction reads indicated in the arc. The graphical simplification of splice junctions was performed using Inkscape.

**Supplementary Figure S4. modified Sashimi plots of 5 nucleotide microexon in *AQP7* gene**

Sashimi plots generated from IGV data of the human SCA3 dataset (n = 6 vs. 6) are shown for (A) affected individuals and (B) controls. A minimum splice junction coverage of five was applied to all samples. For clarity, splice junctions were simplified by merging parallel junctions that skip or include the same microexon into a representative connecting line, with the summed number of junction reads indicated in the arc. The graphical simplification of splice junctions was performed using Inkscape.

**Supplementary Figure S5. modified Sashimi plots of 11 nucleotide microexon in *BLOC1S6* gene**

Sashimi plots generated from IGV data of the human PD dataset (n = 6 vs. 6) are shown for (A) affected individuals and (B) controls. A minimum splice junction coverage of five was applied to all samples. For clarity, splice junctions were simplified by merging parallel junctions that skip or include the same microexon into a representative connecting line, with the summed number of junction reads indicated in the arc. The graphical simplification of splice junctions was performed using Inkscape.

**Supplementary Figure S6. modified Sashimi plots of 16 nucleotide microexon in *CACNB4* gene**

Sashimi plots generated from IGV data of the cellular SCA3 dataset (n = 4 vs. 4) are shown for (A) affected cell lines and (B) controls. A minimum splice junction coverage of five was applied to all samples. For clarity, splice junctions were simplified by merging parallel junctions that skip or include the same microexon into a representative connecting line, with the summed number of junction reads indicated in the arc. The graphical simplification of splice junctions was performed using Inkscape.

**Supplementary Figure S7. modified Sashimi plots of 26 nucleotide microexon in *CHPT1* gene**

Sashimi plots generated from IGV data of the human PD dataset (n = 6 vs. 6) are shown for (A) affected individuals and (B) controls. A minimum splice junction coverage of five was applied to all samples. For clarity, splice junctions were simplified by merging parallel junctions that skip or include the same microexon into a representative connecting line, with the summed number of junction reads indicated in the arc. The graphical simplification of splice junctions was performed using Inkscape.

**Supplementary Figure S8. modified Sashimi plots of 22 nucleotide microexon in *DPH5-DT* gene**

Sashimi plots generated from IGV data of the cellular SCA3 dataset (n = 4 vs. 4) are shown for (A) affected cell lines and (B) controls. A minimum splice junction coverage of five was applied to all samples. For clarity, splice junctions were simplified by merging parallel junctions that skip or include the same microexon into a representative connecting line, with the summed number of junction reads indicated in the arc. The graphical simplification of splice junctions was performed using Inkscape.

**Supplementary Figure S9. modified Sashimi plots of 25 nucleotide microexon in *EGFL7* gene**

Sashimi plots generated from IGV data of the human PD dataset (n = 6 vs. 6) are shown for (A) affected individuals and (B) controls. A minimum splice junction coverage of five was applied to all samples. For clarity, splice junctions were simplified by merging parallel junctions that skip or include the same microexon into a representative connecting line, with the summed number of junction reads indicated in the arc. The graphical simplification of splice junctions was performed using Inkscape.

**Supplementary Figure S10. modified Sashimi plots of 3 nucleotide microexon in *HP1BP3* gene**

Sashimi plots generated from IGV data of the cellular PD dataset (n = 4 vs. 4) are shown for (A) affected cell lines and (B) controls. A minimum splice junction coverage of five was applied to all samples. For clarity, splice junctions were simplified by merging parallel junctions that skip or include the same microexon into a representative connecting line, with the summed number of junction reads indicated in the arc. The graphical simplification of splice junctions was performed using Inkscape.

**Supplementary Figure S11. modified Sashimi plots of 26 nucleotide microexon in *HSD11B1L* gene**

Sashimi plots generated from IGV data of the cellular PD dataset (n = 4 vs. 4) are shown for (A) affected cell lines and (B) controls. A minimum splice junction coverage of five was applied to all samples. For clarity, splice junctions were simplified by merging parallel junctions that skip or include the same microexon into a representative connecting line, with the summed number of junction reads indicated in the arc. The graphical simplification of splice junctions was performed using Inkscape.

**Supplementary Figure S12. modified Sashimi plots of 18 nucleotide microexon in *MRPL33* gene**

Sashimi plots generated from IGV data of the cellular PD dataset (n = 4 vs. 4) are shown for (A) affected cell lines and (B) controls. A minimum splice junction coverage of five was applied to all samples. For clarity, splice junctions were simplified by merging parallel junctions that skip or include the same microexon into a representative connecting line, with the summed number of junction reads indicated in the arc. The graphical simplification of splice junctions was performed using Inkscape.

**Supplementary Figure S13. modified Sashimi plots of 12 nucleotide microexon in *NDUFAF5* gene**

Sashimi plots generated from IGV data of the human SCA3 dataset (n = 6 vs. 6) are shown for (A) affected individuals and (B) controls. A minimum splice junction coverage of five was applied to all samples. For clarity, splice junctions were simplified by merging parallel junctions that skip or include the same microexon into a representative connecting line, with the summed number of junction reads indicated in the arc. The graphical simplification of splice junctions was performed using Inkscape.

**Supplementary Figure S14. modified Sashimi plots of 22 nucleotide microexon in *PABPC1L* gene**

Sashimi plots generated from IGV data of the cellular PD dataset (n = 4 vs. 4) are shown for (A) affected cell lines and (B) controls. A minimum splice junction coverage of five was applied to all samples. For clarity, splice junctions were simplified by merging parallel junctions that skip or include the same microexon into a representative connecting line, with the summed number of junction reads indicated in the arc. The graphical simplification of splice junctions was performed using Inkscape.

**Supplementary Figure S15. modified Sashimi plots of 20 nucleotide microexon in *TENM4* gene**

Sashimi plots generated from IGV data of the human PD dataset (n = 6 vs. 6) are shown for (A) affected individuals and (B) controls. A minimum splice junction coverage of five was applied to all samples. For clarity, splice junctions were simplified by merging parallel junctions that skip or include the same microexon into a representative connecting line, with the summed number of junction reads indicated in the arc. The graphical simplification of splice junctions was performed using Inkscape.

**Supplementary Figure S16. modified Sashimi plots of 13 nucleotide microexon in *TMEM120A* gene**

Sashimi plots generated from IGV data of the human PD dataset (n = 6 vs. 6) are shown for (A) affected individuals and (B) controls. A minimum splice junction coverage of five was applied to all samples. For clarity, splice junctions were simplified by merging parallel junctions that skip or include the same microexon into a representative connecting line, with the summed number of junction reads indicated in the arc. The graphical simplification of splice junctions was performed using Inkscape.

**Supplementary Figure S17. modified Sashimi plots of 5 nucleotide microexon in *TMEM144* gene**

Sashimi plots generated from IGV data of the human PD dataset (n = 6 vs. 6) are shown for (A) affected individuals and (B) controls. A minimum splice junction coverage of five was applied to all samples. For clarity, splice junctions were simplified by merging parallel junctions that skip or include the same microexon into a representative connecting line, with the summed number of junction reads indicated in the arc. The graphical simplification of splice junctions was performed using Inkscape.

**Supplementary Figure S18. modified Sashimi plots of 11 nucleotide microexon in *TOP3A* gene**

Sashimi plots generated from IGV data of the human PD dataset (n = 6 vs. 6) are shown for (A) affected individuals and (B) controls. A minimum splice junction coverage of five was applied to all samples. For clarity, splice junctions were simplified by merging parallel junctions that skip or include the same microexon into a representative connecting line, with the summed number of junction reads indicated in the arc. The graphical simplification of splice junctions was performed using Inkscape.

**Supplementary Figure S19. modified Sashimi plots of 29 nucleotide microexon in *WDR62* gene**

Sashimi plots generated from IGV data of the cellular PD dataset (n = 4 vs. 4) are shown for (A) affected cell lines and (B) controls. A minimum splice junction coverage of five was applied to all samples. For clarity, splice junctions were simplified by merging parallel junctions that skip or include the same microexon into a representative connecting line, with the summed number of junction reads indicated in the arc. The graphical simplification of splice junctions was performed using Inkscape.

**Supplementary Figure S20. Validation of selected microexon events through qPCR in SH-SY5Y cell models.** The relative expression levels of isoform-specific qPCR assays for inclusion and skipping were assessed for *MEF2C* and *MRPL33* in the PD model, as well as *MEF2A* and *PHB2* in the SCA3 model. Each group consisted of four biological replicates, with 2–3 technical replicates per sample. Expression levels were normalized against a target-specific reference amplicon using the 2⁻ΔΔCq method. The data are presented as mean ± SEM, compared to the respective control group.
